# A retrospective study of post-operative complications and cost analysis in robotic rectal resection versus laparoscopic rectal resection

**DOI:** 10.3389/fsurg.2022.969038

**Published:** 2022-08-19

**Authors:** Muhammad Ali, Xiaodong Zhu, Yang Wang, Jianyue Ding, Qi Zhang, Qiannan Sun, Shantanu Baral, Daorong Wang

**Affiliations:** ^1^ Department of Gastrointestinal Surgery, Northern Jiangsu People’s Hospital Affiliated to Yangzhou University, Yangzhou, China; ^2^ General Surgery Institute of Yangzhou, Yangzhou University, Yangzhou, China; ^3^ Medical College of Yangzhou University, Yangzhou, China

**Keywords:** robotic da vinci surgery, laparoscopic surgery, rectal cancer, short-term complication, cost analysis

## Abstract

**Background:**

Robotic rectal cancer surgery has proven to be a viable alternative to laparoscopic surgery in treating rectal cancer. This study assessed the short-term operative measures of robotic versus laparoscopic surgery.

**Material:**

Data was obtained retrospectively from July 2019 to November 2021. Patient demographics, pre-and post-operative features, initial bowel movement, length of hospital stay, and short-term postoperative outcomes such as harvested lymph node, sepsis, Clavien–Dindo Classification, and cost were evaluated.

**Results:**

A total of 155 patients were treated for colorectal cancer, with 64 receiving robotic surgery and 91 receiving laparoscopic surgery. According to the Clavien–Dindo classification, there is a significant *P* < 0.05 between robotic and laparoscopic rectal surgery, with robotic having fewer patients in grade III-IV than laparoscopic. Despite this, laparoscopic surgery is associated with more sepsis patients (*P* < 0.05), and harvested lymph nodes are likewise associated with significant results.

**Conclusion:**

With respect to post-operative complication and cost analysis, our finding imply that robotic rectal resection achieves better-quality short-term outcome but more costly than laparoscopic as well as Clavien–Dindo classification plays a crucial role in assessing postoperative rectal cancer complications and considerably impacts the quality of life.

## Introduction

Colorectal cancer is becoming more common, accounting for around 30% of rectal cancer cases (1). Colorectal carcinoma is a life-threatening condition, and if routine health screenings were not performed, the disease might have progressed to an advanced stage by the time it is diagnosed. If colorectal cancer is identified early enough, surgery may be able to stop it from spreading (2). As technology and operative procedures have advanced, laparoscopic surgery (3) and, more recently, robotic-assisted surgery (4). have become viable treatment options for colorectal cancer. On the other hand, laparoscopic procedures have the drawback of an unsteady picture, which is especially problematic in patients with a narrow pelvis.

Many of the technical limitations of laparoscopy are addressed by robotic-assisted surgery, which provides a three-dimensional field of view, articulating instruments with 7 degrees of motion, the ability to retract and control the camera without the use of an assistant, and the small constraints of the pelvis may allow for more precise dissection. Robotic rectal resection (RRR) is becoming more commonly used in rectal cancer resection because of these benefits (5).

Several studies have been conducted to evaluate the outcome of laparoscopic versus robotic-assisted procedures for treating rectal cancer in terms of functional outcome, both short-term and long-term (6–9). However, the actual benefits of robotic surgery over laparoscopic surgery in terms of post-operative problems are uncertain, and few large-scale trials exist.

In order to assess the impact of the pre-and post-operative short-term outcome, we conducted a retrospective comparison research of RRR and laparoscopic rectal resection (LRR) done at our department.

## Materials and methods

### Study population

Our centre is a government university hospital where several robotic procedures are performed, including colorectal, hepato-biliary, gastro-esophageal, and retro-peritoneal procedures. From July 2019 through November 2021, a database of laparoscopic and robotic rectal procedures was created, and data was prospectively recorded. The institutional review board accepted the study at Northern Jiangsu People's Hospital operations were performed using a Da Vinci Robotic Surgical System model Xi (Intuitive Surgical, Sunnyvale, CA, USA).

All the cases were performed by the same group of surgeons, all of whom have extensive experience with laparoscopic and robotic surgery. Patients were given the choice of open, laparoscopic, or robotic surgery. Robotic surgery has the same indications as open and laparoscopic surgery, regardless of the stage of neoplasia. A few people were obstinate about not pursuing modern technology. An agreement for surgical treatment was achieved during a weekly multidisciplinary conference including surgeons, oncologists, gastroenterologists, and radiologists held every Monday at Northern Jiangsu People's Hospital.

### Inclusion criteria

A verified histological diagnosis of the colorectal malignant tumour was essential for inclusion in this study. Moreover, obtaining the informed consent of the patient for surgical treatment.

### Exclusion criteria

Patients with gastrointestinal bleeding, inflammatory bowel disease, synchronous tumours, benign disorders, and clinical T4 stage tumours that did not respond to the neoadjuvant treatment were excluded from the study.

### Pre-operative study

A physical exam, complete colonoscopy with biopsy, rigid rectoscopy, pelvic magnetic resonance imaging scan (MRI), thorax and abdomen computed tomography (CT), and carcinoembryonic antigen (CEA) measurement were all included in the standardized pre-operative workup for all patients. The tumoral stage was assessed by selecting the T and N of the worst stage revealed by any imaging technology using the TNM staging criteria (American Joint Committee on Cancer).

### Data collected

The primary patient demographic characteristics, including pre-operative TNM stage and distance from the anal verge, tumor size, lymphovascular invasion, Tumor grade, American Society of Anaesthesiologists (ASA) score, Carcinoembryonic Antigen (CEA), and BMI, were assessed and compared retrospectively between the two groups. The main peri-operative and post-operative data, morbidity, and mortality were also evaluated, as were the Total mesorectal excision (TME), distal resection margin (DRM), and proximal resection margin (PRM) harvested lymph node (HLN), and Clavien–Dindo Classification (CDC). When there was a clinical suspicion of anastomosis leaking, it was diagnosed (change in drainage, fever, or abdominal pain). A contrast enema detected during a control CT was always used to confirm this diagnosis. Up to 90 days after surgery, data on hospital stay and readmission were obtained.

### Post-operative follow-up

The post-operative results included operative time, estimated blood loss, time until flatus passage, liquid diet duration, length of postoperative hospital stay, and total cost. The operative time was defined as the time between making a skin incision and closing it and the time required for robotic surgery docking and undocking. When patients complained of their initial bouts of flatulence, they were put on a liquid diet. The length of time spent in the hospital after surgery was defined as the time between surgery and discharge. Surgery, anesthetic, drugs, and care were all included in the entire cost.

### Statistics

The data was analyzed using SPSS version 25.0 (SPSS, Inc., Chicago, Illinois). Continuous variables were represented by their mean and standard deviation, whereas categorical variables were represented by their number (percentage). The qualitative and quantitative differences between subgroups were analyzed using the Chi-Squared test for categorical components and the student *t*-test for continuous parameters. Statistical significance was defined as a *P* < 0.05.

## Results

There was a total of 64 RRR and 91 LRR in this study. In both groups, the patient demographics and pre-operative features were similar. There was no significant difference in tumour size, distance from the anal verge, and lymphovascular invasion between the two groups. BMI shows a statistically significant result of *P* < 0.05 (see Table 1).

**Table 1:** Patient's demographic characteristics.

Rf.	Laparoscopic (*n *= 91)	Robotic (*n *= 64)	*P*-value
Gender			0.856
Male [*n* (%)]	59 (64%)	40 (62.5%)	
Female [*n* (%)]	32 (35%)	24 (37.5%)	
Age [*n* (SD)]	62.67 (11.2)	61.7 (12.04)	0.590
BMI (kg/m^2^) [*n* (SD)]	24.34 (2.32)	24.4 (3.36)	0.015
CEA (U/ml) [*n* (SD)]	3.6 (2.3)	3.2 (2.08)	0.24
Comorbidity [*n* (%)]			
CVS	38 (41.7%)	30 (46.8%)	0.622
Diabetes	8 (8.8%)	10 (15.6%)	0.211
Pulmonary disease	28 (30.7%)	30 (46.8%)	0.391
Kidney disease	15 (16.4%)	10 (15.6%)	0.256
Neurocognitive disease	5 (5.5%)	0 (0%)	0.077
Intestinal obstruction	0 (0%)	3 (4.7%)	0.070
Previous abdominal surgery	15 (16.4%)	17 (26.5%)	0.159
Tumour grade [*n* (%)]			0.127
Well differentiated	8 (8.8%)	3 (4.7%)	
Moderately differentiated	60 (66%)	35 (54.6%)	
Poorly differentiated	23 (25%)	25 (39%)	
Mucinous	0 (0%)	1 (1.5%)	
ASA score [*n* (%)]			0.352
1	17 (18.6%)	7 (11%)	
2	52 (57%)	38 (59%)	
3	22 (24%)	18 (28%)	
4	0 (0%)	1 (1.5%)	
TNM staging [*n* (%)]			
T1	5 (5.5%)	4 (6.2%)	1
T2	17 (18%)	18 (19.7%)	0.178
T3	23 (25%)	19 (20.8%)	0.585
T4	43 (47%)	22 (24%)	0.137
Tumor distance from anal verge (cm) [*n* (SD)]	7.89 (3.9)	8.24 (4.2)	0.431
Tumor size (cm) [*n* (SD)]	4.95 (1.67)	4.72 (1.85)	0.339
Lympho-vascular invasion (cm) [*n* (SD)]	2.1 (1.3)	2.7 (1.4)	0.509

CEA, carcinoembryonic antigen; BMI, body mass index; CVS, cardiovascular system; RBC, red blood cell; WBC, white blood cell; ASA, American Society of Anaesthesiologists.

The primary operative data are depicted in Table 2. The RRR group had a much longer operating time (mean: 177 min) than the LRR group (mean: 162 min) (*P* = 0.361). Mean intra-operative estimated blood loss shows significant results *P* < 0.05, and conversion rates *P* = 1.

**Table 2:** Intra-operative and pathology data.

Intra-operative	Laparoscopic (*n *= 91)	Robotic (*n *= 64)	*P*-value
Operative time (min) [*n* (SD)]	162 (46)	177 (42)	0.361
Conversion to laparotomy [*n* (%)]	2 (2.2%)	1 (1.5%)	1
Operative blood loss (ml) [*n* (SD)]	59.9 (26.4)	48.9 (21.7)	<0.001
No. of transfused Patient [*n* (%)]	13 (14.2%)	15 (23.4%)	0.202
Surgical procedure [*n* (%)]			0.030
Dixon	40 (44%)	32 (50%)	
Dixon & fistulation	17 (18.6%)	21 (32.8%)	
Hartmann	9 (9.9%)	2 (3.1%)	
Miles	25 (27.4%)	9 (14%)	
Clavien–Dindo classification [*n* (%)]			<0.001
I	19 (21%)	18 (28%)	
II	8 (8.8%)	17 (26%)	
III	58 (63.7%)	28 (43%)	
IV	8 (8.8%)	0 (0%)	
HLN (*n*) [*n* (SD)]	12.47 (5.48)	12.55 (7.32)	0.027
DRM (cm) [*n* (SD)]	4.08 (1.44)	4.40 (0.91)	<0.001
PRM (cm) [*n* (SD)]	10.84 (1.3)	9.92 (0.630)	<0.001

GERD, gastroesophageal reflux disease; HLN, harvested lymph node; DRM, distal resection margin; PRM, proximal resection margin.

The Clavien–Dindo grading system exposes a substantial *P* < 0.05, whereas grade III–IV patients are seen more frequently in laparoscopic surgery than robotic surgery. The results of the distal and proximal resection margins are statistically significant, *P* < 0.05. The lymph nodes that were harvested similarly showed a significant result (*P* < 0.05) (see Table 2).

The mean number of hospital stay days is statistically significant at *P* < 0.05, and neither group had any instances of hospital mortality. Patients in the RRR group had a shorter period of flatus than those in the LRR group (mean, 3.4 vs. 4.5 days, *P* = 0.5). Return to diet was not significantly different in both groups (mean, 4.8 vs. 5.5 days, *P* = 0.8). Overall, the RRR group had 33% of the complications, compared to 46% in the LRR group *P* = 0.101. The RRR group had a 0% overall rate of anastomotic leakage, while the LRR group had a 1% rate. Sepsis has increased by 3.2% in the LRR group, while it has decreased by 1.5% in the RRR group *P* = 0.63 (see Table 3). The total cost of hospital stays as well as the cost of operation reveal a significant result *P* < 0.05, with robotic surgery being significantly more expensive than laparoscopic surgery (see Table 4).

**Table 3:** Postoperative outcome.

Outcome	Laparoscopic (*n *= 91)	Robotic (*n *= 64)	*P*-value
Hospital stays days [*n* (SD)]	15.70 (4.52)	14.72 (10.45)	0.008
Time of flatus (Days) [*n* (SD)]	4.28 (1.43)	3.44 (1.7)	0.523
Return to diet (Days) [*n* (SD)]	5.53 (1.7)	4.80 (2.6)	0.855
Pt. with post-operative complication [*n* (%)]	42 (46%)	21 (33%)	0.101
Cough [*n* (%)]	12 (13%)	5 (7.8%)	0.314
Fever [*n* (%)]	22 (24%)	11 (17%)	0.325
Anastomotic leakage [*n* (%)]	1 (1%)	0 (0%)	1
Wound infection [*n* (%)]	1 (1%)	0 (0%)	1
Retention of urine [*n* (%)]	1 (1%)	0 (0%)	1
GERD [*n* (%)]	0 (0%)	1 (1.5%)	0.413
Sepsis [*n* (%)]	3 (3.2%)	1 (1.5%)	0.643
Re-operation [*n* (%)]	2 (2.2%)	2 (3.1%)	1

GERD, gastroesophageal reflux disease; HLN, harvested lymph node; DRM, distal resection margin; PRM, proximal resection margin; TME, total Meso-rectal excision.

**Table 4:** Cost.

Variables	Laparoscopic *n* (SD)	Robotic *n* (SD)	Mean difference	*P-*value
Surgical cost (RMB)	6,296 (819)	7,854 (3,688)	1,558	<0.001
Instrument cost (RMB)	18,774 (4,746)	39,333 (8,576)	20,559	0.029
Total cost (RMB)	55,454 (7,931)	73,499 (11,532)	18,045	0.006

n, Mean; SD, Standard Deviation.

## Discussion

We examined the operating time, intra-operative blood loss, time to the passing of flatus, liquid diet duration, total cost, and length of postoperative hospital stay to assess the feasibility and short-term postoperative outcomes in Clavien–Dindo grading, sepsis, anastomotic leakage, and harvested lymph node of RRR and LRR.

In this study, RRR demonstrated a trend of longer operating times than LRR, but the difference was not significant. According to several retrospective investigations, RRR, on the other hand, takes longer to operate than LRR (10–12). The extended operative timeframes for RRR may have been influenced by the learning curve period, which was overlooked in previous analysis. Furthermore, according to a thorough analysis, robotic surgery's operative times have improved, and after 41 instances, it was faster than laparoscopy (13).

The expected intraoperative blood loss during RRR was lower than LRR. Hence intraoperative blood loss was determined by subtracting aspirated and given fluids. Three other RCTs have already reported comparable findings (14–16). The RRR group had a significantly lower conversion rate than the LRR group, consistent with previous results (12–16). Two instances in the LRR group required an open surgical approach due to difficult mesorectal dissection. Peritoneal adherence and pneumoperitoneum intolerance were factors in one conversion in the RRR group.

The RRR group recovers bowel function faster than the LRR group, and the LRR group's hospital stay is longer than the RRR group. Our findings are consistent with those of the prior study (17–19). Post-operative problems may impact longer hospital admissions in LRR, so we evaluate short-term 90-day outcomes.

We analyzed the complication according to the Clavien–Dindo classification. It allows us to evaluate the surgical outcomes in medical practice, and this is a simple, objective, reproducible, and valuable tool for assessing postoperative advancement around the world. We focused on grade III–IV post-operative complications since they are the most difficult to manage for improving quality of life, clinical support, and survival. However, in our research, we found a significantly lower RRR rate.

There were significant differences in the lymph node yield between the two groups, with the RRR group having an average of 12.55 lymph nodes removed with *P* < 0.05. In 1990, the World Congress of Gastroenterology in Sydney recommended the removal of twelve lymph nodes (20). However, a long-term follow-up is required for a fair assessment of oncological outcomes connected to RRR.

According to our findings, anastomotic leaking is more common in laparoscopic surgery than robotic surgery. The 3D dimension and articulating equipment used in robotic surgery may make anastomosis easier. Sepsis is also reported in LRR, which could be related to a lack of cleanliness due to a higher number of surgeon assistants than in Da Vinci. It could be due to camera control in LRR, which necessitates numerous removals from the body to clean it.

In our study, RRR had higher overall hospital expenses than LRR. RRR is more expensive than previously anticipated, according to several research (21, 22). A cost discrepancy may be possible due to RRR's complex instrument handling.

The capacity to diminish intra-operative gastro-intestinal stimulation and promote gastro-intestinal function recovery, which would block intestinal function recovery, is one of the possible benefits of using robots in colorectal surgery. Additionally, faster digestive function recovery reduces water and electrolyte imbalances, postoperative intestinal adhesion and other problems, patient rehabilitation, and hospital stay.

This is the only study we know that directly compares postoperative outcomes. However, there are several limitations. (1) A retrospective study was done over a brief period. (2) The study has little data. (3) There is no thought given to long-term effects. (4) The best long-term survival outcome will ultimately depend on further extensive clinical research on postoperative problems.

## Conclusion

Compared to laparoscopic rectal cancer, RRR is the most practicable and high-quality approach for rectal carcinoma, with better surgical results in Clavien–Dindo grade III–IV, sepsis, harvested lymph nodes, and intra-operative blood loss, but more costly than laparoscopic. However, additional clinical trials are required to validate it. Furthermore, long-term oncological implications must be a primary focus of future research.

## Data Availability

The original contributions presented in the study are included in the article/Supplementary Material, further inquiries can be directed to the corresponding author.
